# On the robustness of an eastern boundary upwelling ecosystem exposed to multiple stressors

**DOI:** 10.1038/s41598-021-81549-1

**Published:** 2021-01-21

**Authors:** Ndague Diogoul, Patrice Brehmer, Hervé Demarcq, Salaheddine El Ayoubi, Abou Thiam, Abdoulaye Sarre, Anne Mouget, Yannick Perrot

**Affiliations:** 1 Institut Sénégalais de Recherches agricoles (ISRA), Centre de Recherches Océanographiques de Dakar-Thiaroye (CRODT), Dakar, Senegal; 2 IRD, CNRS, Univ Brest, Ifremer, DR Ouest, Lemar, Plouzané, France; 3 IRD, IFREMER, CNRS, Univ Montpellier, Marbec, Sète, France; 4 Institut National de Recherche Halieutique INRH, Agadir, Morocco; 5 University Cheikh Anta Diop (UCAD), Institute of Environmental Science (ISE), BP 5005, Dakar, Senegal

**Keywords:** Ecology, Ocean sciences

## Abstract

The resistance of an east border upwelling system was investigated using relative index of marine pelagic biomass estimates under a changing environment spanning 20-years in the strongly exploited southern Canary Current Large marine Ecosystem (sCCLME). We divided the sCCLME in two parts (north and south of Cap Blanc), based on oceanographic regimes. We delineated two size-based groups (“plankton” and “pelagic fish”) corresponding to lower and higher trophic levels, respectively. Over the 20-year period, all spatial remote sensing environmental variables increased significantly, except in the area south of Cap Blanc where sea surface Chlorophyll-a concentrations declined and the upwelling favorable wind was stable. Relative index of marine pelagic abundance was higher in the south area compared to the north area of Cap Blanc. No significant latitudinal shift to the mass center was detected, regardless of trophic level. Relative pelagic abundance did not change, suggesting sCCLME pelagic organisms were able to adapt to changing environmental conditions. Despite strong annual variability and the presence of major stressors (overfishing, climate change), the marine pelagic ressources, mainly fish and plankton remained relatively stable over the two decades, advancing our understanding on the resistance of this east border upwelling system.

## Introduction

The Canary Current Large Marine Ecosystem (CCLME) is the largest eastern boundary upwelling ecosystem (EBUE) globally. It is ranked second to third in terms of primary productivity globally^1^, and is of major economic and social importance in providing sustainable livelihoods, fish-protein supplies, and revenue for the coastal populations and states of West African countries. Because upwelling-favorable winds (UW) have different seasonal dynamics along the coast, important environmental differences exist in different parts of the CCLME^2^. The upwelling is permanent in the central part of the system (∼ 21–26°N), and is less intense and more variable off Morocco (26–33°N), peaking in summer. Upwelling is seasonal from Guinea to Senegal (10–16°N). Finally, it gradually changes from winter to spring in the productive season between Mauritania and Senegal (16–21°N)^3^. The seasonality of the upwelling in this region is associated with seasonal movements of the intertropical convergence zone (ITCZ), which drives latitudinal changes in the trade winds^4,5^. The wind-forced upwelling of deep, nutrient-rich water is responsible for high phytoplankton primary productivity in coastal waters^6^, which provides food for higher trophic levels.

Marine organisms, including phytoplankton to small pelagic fishes, are sensitive to environmental changes^7^. For many fish species, the interaction between fishing and climate impacts the life history parameters of populations (growth, maturation, recruitment), migration, spatial distribution, and food web complexity and stability^8^. The fisheries of certain pelagic fish species, such as the Atlantic herring (*Clupea harengus*) and sardine (*Sardina pilchardus*), have fluctuated for many hundreds years, frequently exhibiting alternating population dominance^9,10^. Previous studies conducted off the southern part of the CCLME ‘sCCLME’ (Sahara Bank to South Senegal) highlighted changes to the abundance and distribution limits of a number of fish species in relation to environmental changes, such as cooling or warming waters^11,12^. Long-term changes to zooplankton and micronekton abundance and distribution have, however, received less focus in the CCLME. Yet, these components of the ecosystem are highly sensitive to environmental changes (e.g., nutrient concentration, salinity, oxygen and temperature)^13,14^. Because of the short life cycles of these species, intermediate communities (zooplankton and micronekton) respond rapidly to mesoscale physical processes associated with currents and frontal structures linked to coastal upwellings^13–15^. Zooplanktonic and micronektonic compartments are essential for sustaining organisms higher up the food web^16–18^; consequently, changes to their community could impact the whole marine ecosystem^19^.

**Figure 1:**
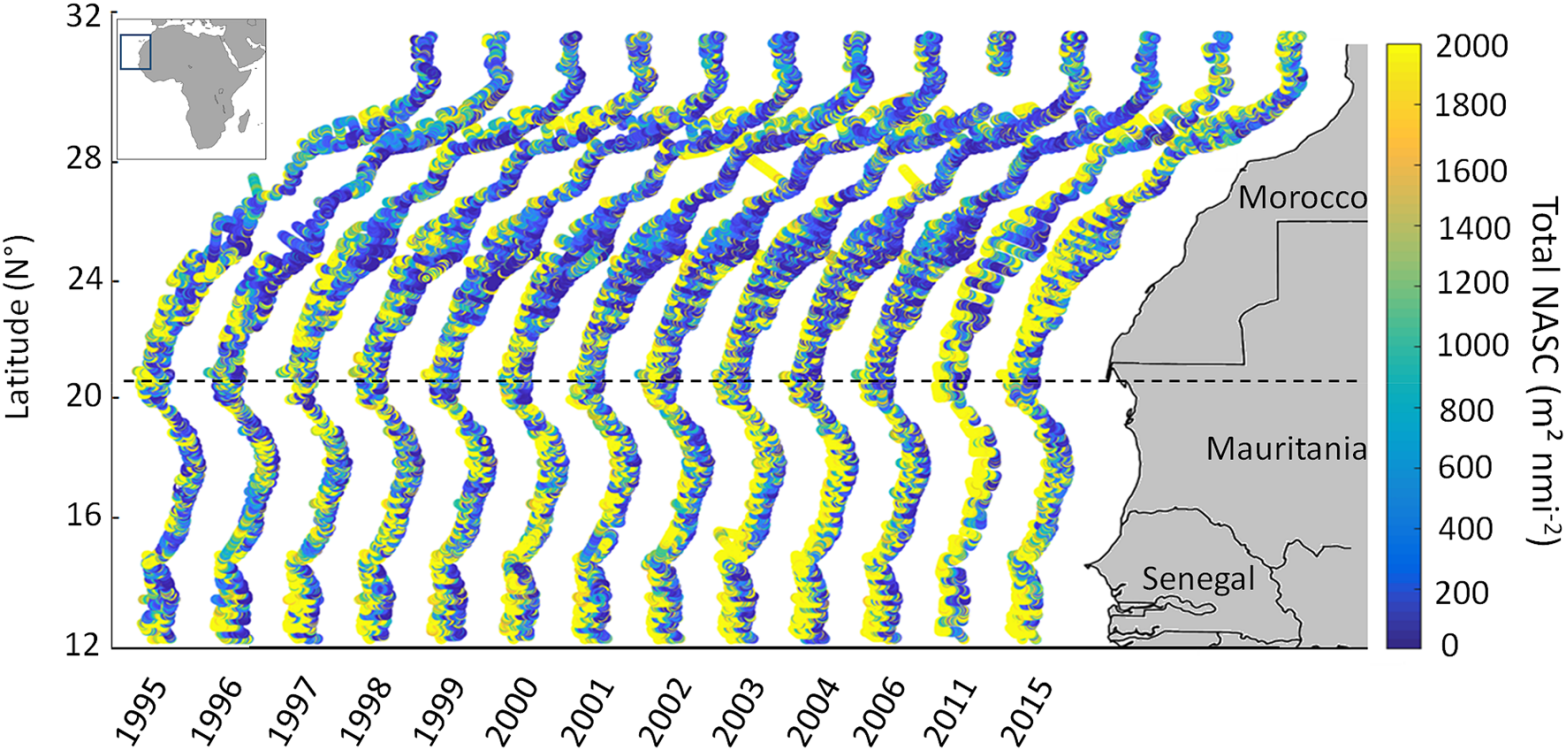
Study area was limited to the continental shelf of the south part of the Canary Current Large Marine Ecosystem. The dashed line represent the Cap Blanc limit dividing the study area into two part. We conductedpelagic acoustic sea survey on the same vessel and used the same protocol to process the total acoustic density of the Nautical Area Scattering Coefficient (NASC, m^2^ nmi^−2^) from 1995 to 2015, as a proxy of pelagic abundance. The map was performed with Matlab R 2018a^20^ (https://fr.mathworks.com/products/new_products/release2018a.html).

This study investigated the resistance in pelagic zooplankton and fish biomass to multiple environmental and anthropomorphic stressors. For instance, in our case, this mainly included overfishing, warming water, and marine pollution. We conducted annual acoustic sea surveys in the sCCLME and compiled associated remote spatial environmental time series (Fig. 1). We then analyzed long-term variability to relative acoustic density used as a proxy of marine pelagic abundance, which was separated into a low and a high trophic group, in relation to environmental factors (i.e., relative abundance, spatial distribution, and diel vertical migration) as specific functional response, from 1995 to 2015. The decision makers (economic, political and administrative authorities in fisheries management) need scientific advices on ecosystem resistance capabilities to counteract the effect of global change and anticipate future change, but such information is difficult to provide in the countries the less developed. Here we propose an original approach taking advantage of the longest times series in the area.

## Results

### Environmental trends

**Figure 2:**
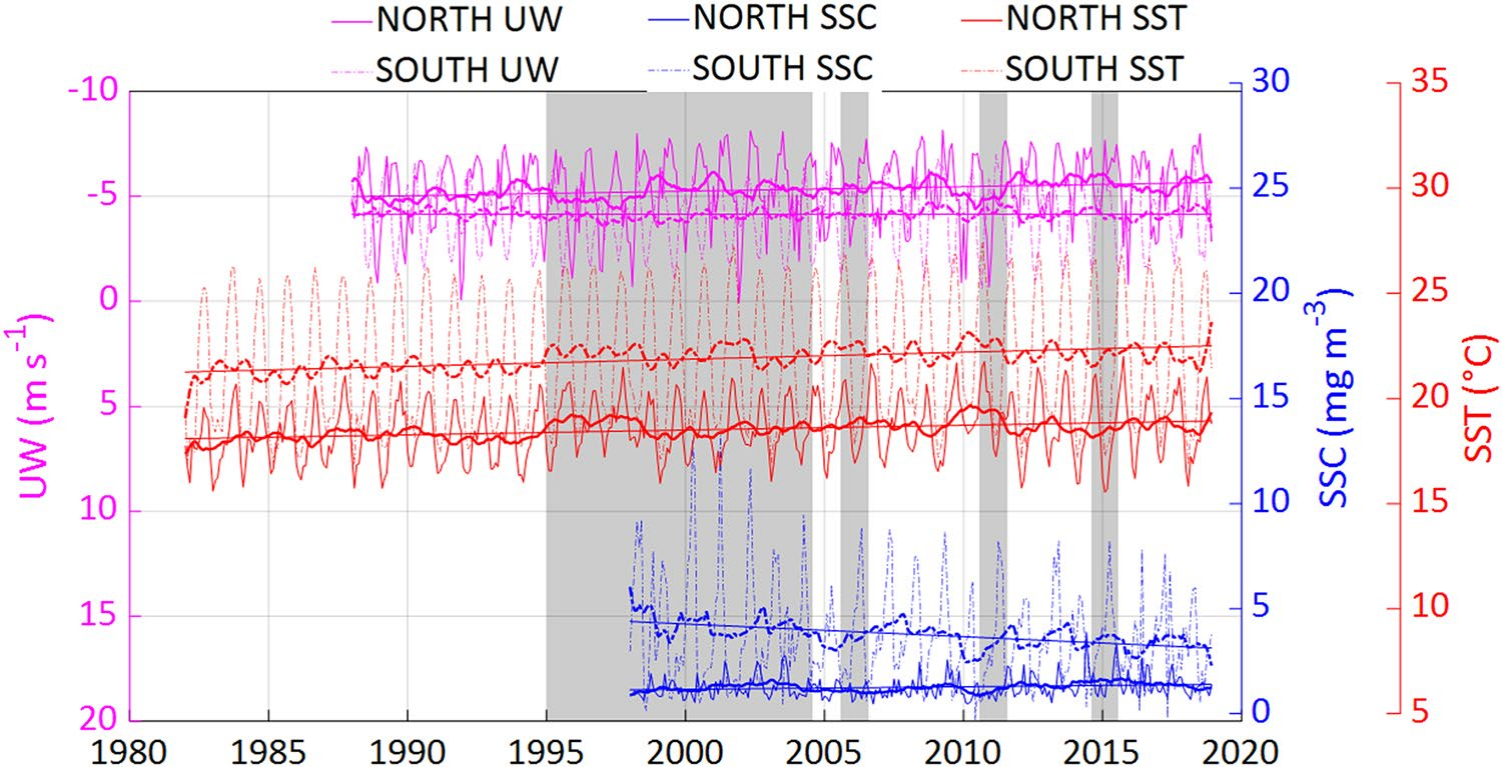
Monthly means and 13-term smoothing average of sea surface temperature (SST, °C), sea surface chlorophyll-a concentration (SSC, mg m^−3^) and upwelling wind (UW, m s^−1^), processed for the areas to the north of Cap Blanc (12–21°N, plain line) and south of Cap Blanc (21–30°N, dashed line). The periods covered by the sea surveys (1995 to 2004, 2006, 2011 and 2015) are shaded in grey.

Analysis of environmental trends showed a significant increase (Table 1) for all variables to the north of Cap Blanc. In comparison, only sea surface temperature (SST) significantly increased to the south of Cap Blanc (Fig. 2).

**Table 1:** Spearman tests for oceanographic environmental trends (favorable Upwelling Wind ‘UW’, Sea Surface Temperature ‘SST’ and Sea Surface Chlorophyll ‘SSC’) from 1995 to 2015 in North and South Cap Blanc.

Area	SST	UW	SSC
	r (*p*-value)	r (*p*-value)	r (*p*-value)
North Cap Blanc	0.5 (**0.001**)	0.1 (**0.02**)	0.4 (**0.001**)
South Cap Blanc	0.6 (**0.001**)	0.006 (0.9)	− 0.6 (**0.001**)

r: temporal linear trend in the Spearman correlation coefficient. Significant values (*p*-value < 0.05) are shown in bold.

**Figure 3:**
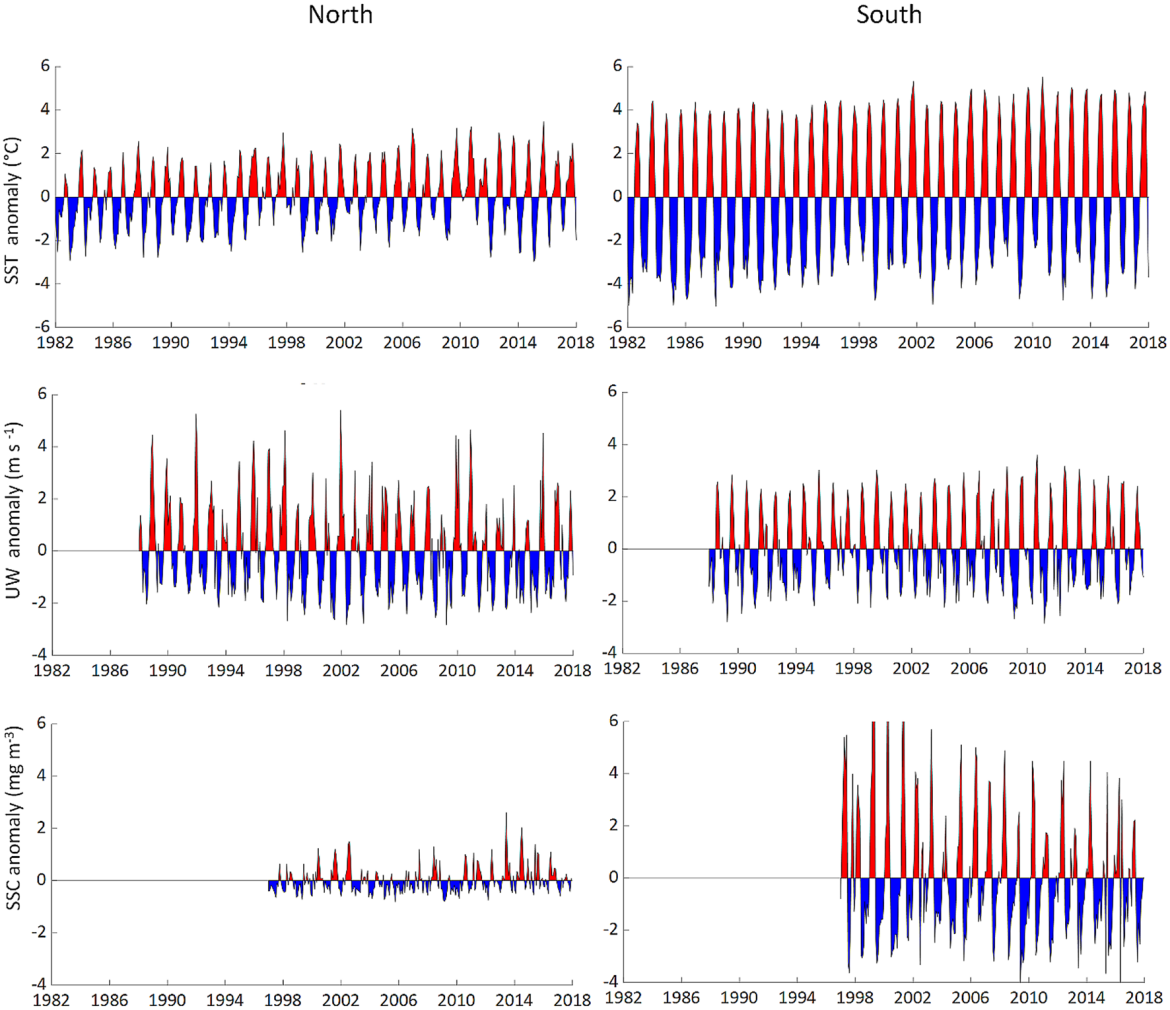
Monthly mean anomalies for (top) sea surface temperature (SST, °C); (middle) favorable upwelling wind (UW, m s^−1^), and (down) sea surface chlorophyll-*a* concentration (SSC, mg m^−3^) over the south part of the Canary Current Large Marine Ecosystem, split into the areas north of Cap Blanc (left) and south of Cap Blanc (right).

SST showed warming trends in both areas of sCCLME, but was stronger in the south (Fig. 2, Table 1). These increasing SST trends were associated with a shift since 1995 (Fig. 2). Over the 20-year study period, SST shifted by 0.65 °C, and 0.75 °C in the north and south, respectively (Fig. S1). To the north of Cap Blanc, consistent successions of negative and positive SST anomalies (± 0.2 °C) were recorded along the time series (Fig. 3). The same SST anomaly pattern was recorded in the south of Cap Blanc, but with higher values (reaching ± 0.5 °C).

The upwelling wind (UW) showed a significant increasing trend in the area north of Cap Blanc, but remained relatively stable in the south (Fig. 2, Table 1). UW shifted similar to SST in the area north of Cap Blanc (Fig. S1). In the area south of Cap Blanc, UW did not show a temporally uniform trend. It decreased from 1998 to 1999, followed by a period of stability (Fig. 2). A succession of negative and positive UW anomalies was documented in the two areas. Positive anomalies were higher in the north, reaching 5 m s^−1^. In comparison, negative anomalies were around 2 m s^−1^ in the north and south of Cap Blanc (Fig. 3).

Sea surface chlorophyll-*a* concentration (SSC) showed the opposite pattern, with a significant increase in the north sCCLME and a very significant decrease in the south (Fig. 2, Table 1). Low SSC positive (+ 2 mg m^3^) and negative (< − 1 mg m^3^) anomalies were recorded in the north of Cap Blanc. In comparison, in the south, high positive (+ 9 mg m^3^) and negative (− 4 mg m^3^) anomalies were recorded.

### Barycenter displacement analysis

**Figure 4:**
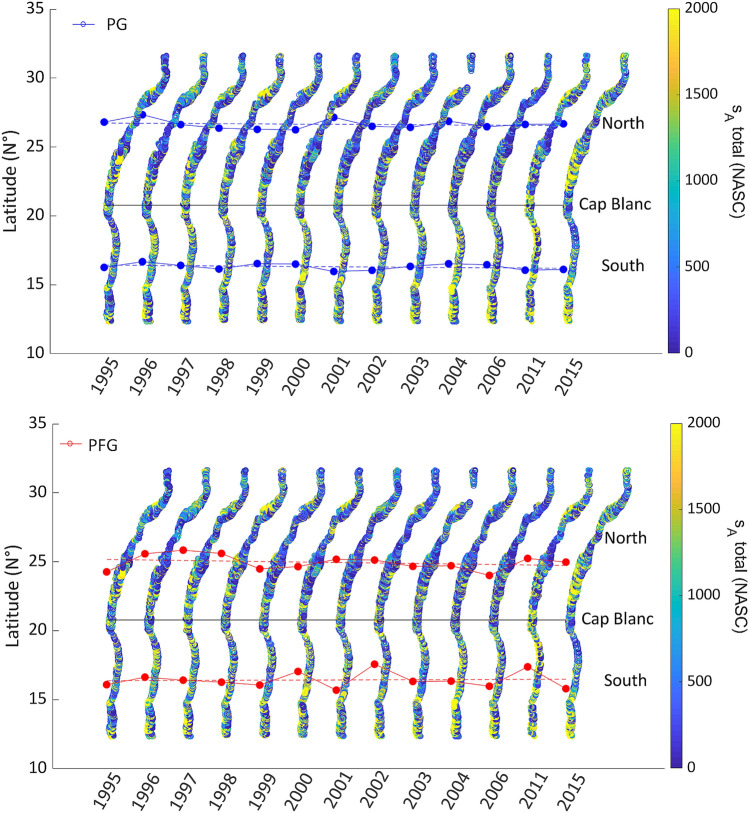
Differences in the two dimensional horizontal barycenter of the two trophic levels studied: (top) zooplankton, micronekton, and ichthyoplankton (named plankton group (PG), blue dots), and (down) pelagic fish and other higher pelagic trophic levels (named pelagic fish group (PFG), red dots). Dashed lines represent the linear trends for each group.

No significant trends were detected for the barycenter of the acoustic density (expressed in Nautical Area Scattering Coefficient (NASC or s_A_, m^2^ nmi^−2^)), which was used as a proxy of pelagic abundance ‘PA’, for both groups in the acoustic surveys (i.e., plankton group (PG) and pelagic fish group (PFG)) from 1995 to 2015 (Fig. 4). The barycenter of PA showed strong inter‐annual fluctuations, particularly in the area south of Cap Blanc where the position of the PG and PFG barycenters was highly variables, sometimes overlapping. There was no significant displacement for both groups in both surveyed areas (north and south of Cap Blanc).

### Analysis of pelagic abundance and anomaly trends

**Figure 5:**
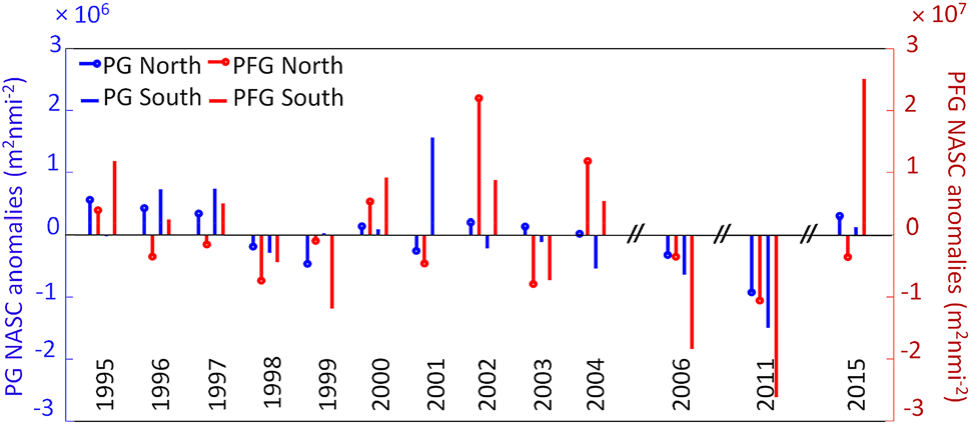
Anomaly of the pelagic mean pelagic abundance (expressed as Nautical Area Scattering Coefficient ‘NASC’, m^2^ nmi^−2^) for the plankton group (PG) and pelagic fish group (PFG) in the areas north and south of Cap Blanc.

PA were higher for PFG compared to PG, with a quantitative ratio of 1/10. PA was higher in the south compared to the north of Cap Blanc for both groups (Fig. 5). No significant trend was observed in PA during the study period (Table 2, *p*-value > 0.05) in the sCCLME. PA was particularly high in 2015 in the area south of Cap Blanc for PFG, whereas abundance dropped by 50% in the north (Fig. 5, Fig. S2). PG showed more stability over the long-term. High positive PA anomaly (> 2 × 10^7^ m^2^ nmi^−2^) was observed for PFG in 2002 in the area north of Cap Blanc. High negative anomalies (> − 2 × 10^7^ m^2^ nmi^−2^) were also recorded in 2006 and 2011 in the area south of Cap Blanc for PFG. For PG, a high positive anomaly (~ 1.5 × 10^6^ m^2^ nmi^−2^) was recorded in 2001 in the area south of Cap Blanc, while a high negative anomaly (~ − 1.5 × 10^6^ m^2^ nmi^−2^) was recorded in 2011. The total of both groups (TG) had the same characteristics as PFG, in terms of variation and anomalies (Fig. S3), due to the weak contribution of PG *vs*. PFG.

**Table 2:** Spearman trend tests for the barycenter displacement of pelagic abundance and Δ_DVM_ trend (i.e., differences between day and night pelagic abundance for the plankton group (PG) and pelagic fish group (PFG)) in the areas north and south of Cap Blanc.

Area	Barycenter trends		Acoustic class trends		Δ_DVM_ trend	
	PG	PFG	PG	PFG	PG	PFG
North Cap Blanc	− 0.04 (0.9)	0.14 (0.6)	− 0. 5 (0.08)	− 0.3 (0.33)	0.4 (0.2)	0.3 (0.3)
South Cap Blanc	− 0.24 (0.5)	0.23 (0.5)	− 0.4 (0.1)	− 0.2 (0.52)	− 0.03 (0.9)	− 0.3 (0.4)

r: Spearman correlation coefficient is shown in the first row of each cell and the *p*-value is shown in parentheses.

### Diel vertical migration (DVM)

**Figure 6:**
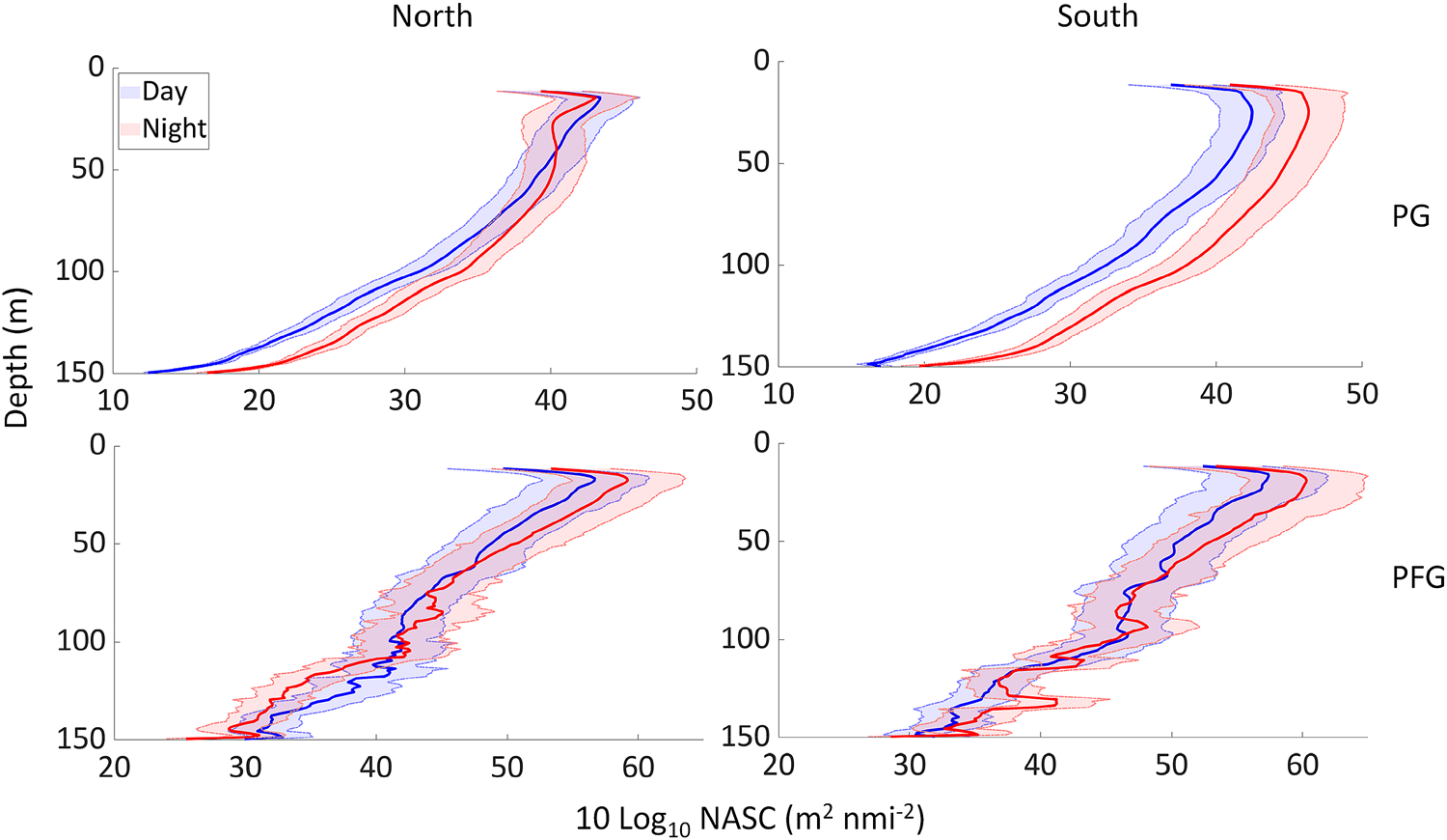
Effect of diel vertical migration (DVM) on the pelagic abundance within the water column [expressed in Nautical Area Scattering Coefficient (m^2^ nmi m^−2^)] on the trends for the plankton group (PG) and pelagic fish group (PFG) in the areas north (left) and south of Cap Blanc (right) in the Canary Current Large Marine Ecosystem. Shaded areas represent the standard deviation.

**Figure 7:**
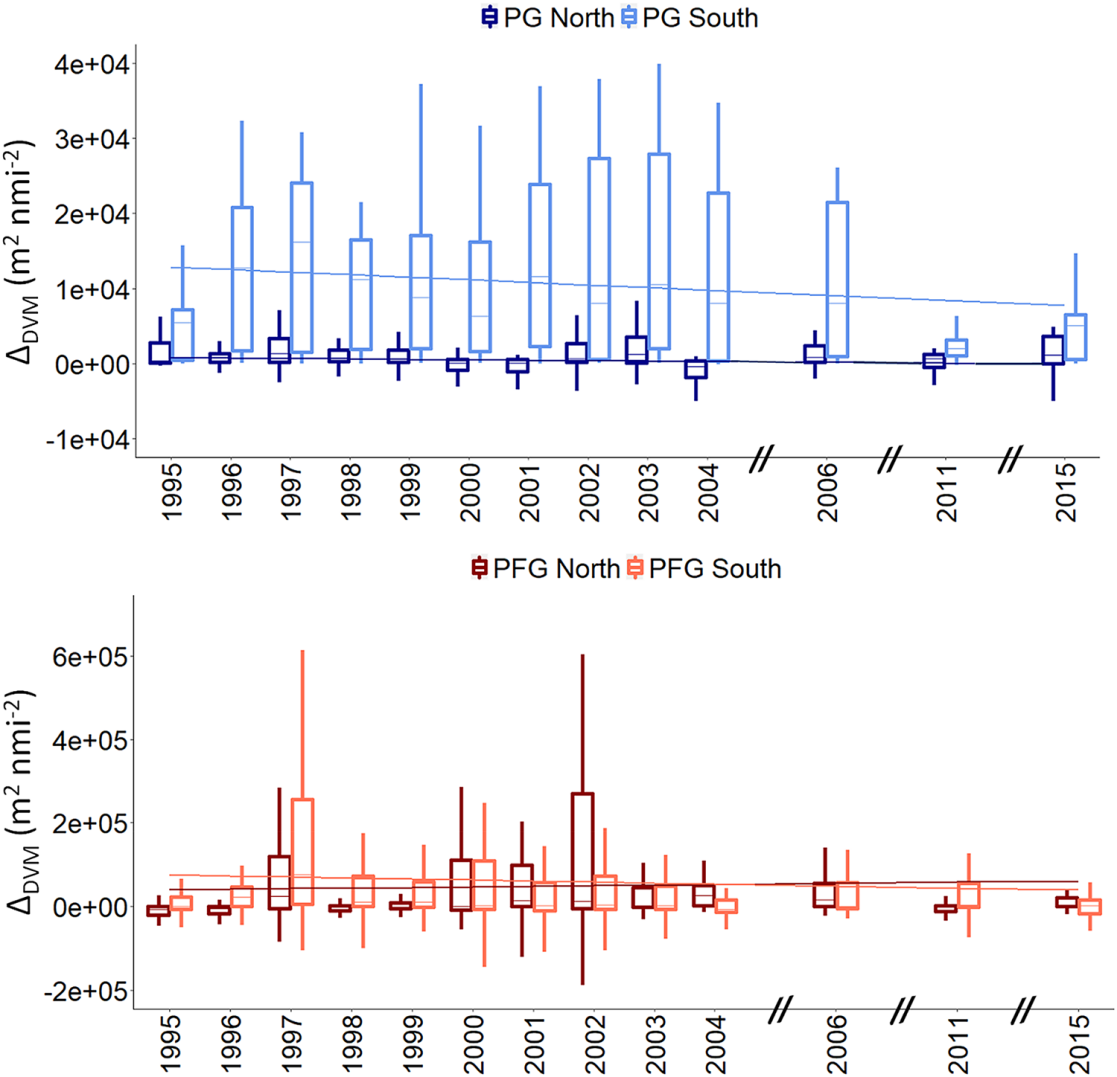
Differences to day and night acoustic density (Delta DVM, expressed in Nautical Area Scattering Coefficient noted NASC or s_A_, m^2^ nmi^−2^) for (top) the plankton group and pelagic fish group (bottom) echo classes in the areas north and south of Cap Blanc.

Diel changes were recorded for all acoustic group in the sCCLME (Figs. 6, 7, Fig. S4). For PG, DVM was normal, with greater PA at night, particularly at the surface (Figs. 6, 7) except in the north of Cap Blanc where the DVM was inversed (i.e., higher PA during the day in the upper water column, 10–40 m). For PFG, PA was also higher at night in the surface waters of both areas. The day-night difference in PA was significant (*p*-value < 0.000) for all groups (Table 3). Δ_DVM_ (i.e., night PA–day PA) mainly produced values, with higher NASC densities at night compared to by day (Fig. 7). Δ_DVM_ was higher in the south compared to the north of Cap Blanc for both acoustic groups, and mostly for PG.

**Table 3:** Wilcoxon test comparing day and night differences in the acoustic abundance of the plankton group (PG) and pelagic fish group (PFG) in the areas north and south of Cap Blanc.

Area	PG	PFG
	w (*p*-value)	(*p*-value)
North Cap Blanc	3 (**< 0.001**)	0 (**< 0.001**)
South Cap Blanc	0 (**< 0.001**)	5 (**< 0.001**)

w: Wilcoxon rank correlation coefficient; Significant values (p-value < 0.05) are shown in bold.

The DVM trend was also evaluated over the 20 years using Δ_DVM_, and no significant trends were found (Table 2). Significant DVM day and night differences were reported in both areas for all groups (Table 3).

## Discussion

In recent years, many changes or trends have been documented in physical forcing at regional and global scales^21^. Most large marine ecosystems (LME) were subjected to continuous warming between 1982 and 2006^22^. A mean warming trend was detected over the last three decades in the whole CCLME region, with substantial warming since 1995^12,23^. Previous study^24^ detected significant SST (*p* < 0.001) warming trends in the CCLME from 1993 to 2014, with markedly higher SST in the southern part (around 0.4 °C decade^−1^) compared to the northern part (about 0.2 °C decade^−1^). Our results support these previous findings, whereby SST warming in the sCCLME was stronger in the area south of Cap Blanc. Bakun^25^ hypothesized that, in the context of the climate change, global warming will enhance land–sea temperature gradients, enhancing UW. We found that UW significantly increased in the area north of Cap Blanc (5.5 m s^−1^ decade^−1^), whereas no UW trend was recorded in the south. Benazzouz et al.^26^ documented the same pattern, with UW strongly increasing between 26°N and 33°N from 1982 to 2011, with this trend being much weaker between 21°N and 26°N. Previous studies demonstrated that warming oceans, including in the CCLME, are associated with a decline in average chlorophyll-*a* concentration ‘Chl-*a*’, which is used as proxy of phytoplankton biomass and primary productivity^27–29^. This declining trend might be explained by a physically mediated effect of upper-ocean warming on vertical stratification, which indirectly affects phytoplankton by limiting nutrient supply to the sunlit layer^28^. It might also be explained by the positive effect of warming on the metabolic rates of plankton^30^. Previous observations in the CCLME indicated that Chl-*a* declined during 1998–2007^31,32^. In other areas, there is evidence that increasing wind speeds overcome any potential increase in stratification, due to warming, increasing Chl-*a* production^33^. In our study, SSC, which was used as a proxy for Chl-*a*, increased significantly in the area north of Cap Blanc, and decreased in the south. Spatial variation in UW and SSC was consistent in the north of Cap Blanc, where increasing SSC coincided with increasing UW. However, in the area south of Cap Blanc, SSC and UW trends were not spatially correlated. Indeed, coastal upwellings depend on a combination of wind intensity, coastal topographic effects, and alongshore geostrophic flow (related to large-scale circulation patterns)^2^. Moreover, there is a non-monotonic relationship between UW and biological responses, where strong UW limits productivity and phytoplankton levels^34,35^. Moderate wind speed provides optimal conditions for the development of coastal phytoplankton populations^36,37^.

The PA is 10 times greater for PFG compared to PG, supporting existing studies suggesting that there is a factor of 10 between fish and zooplankton biomass in the marine food web^38^. Marine ecosystems have generally inverted biomass pyramids^39,40^ with the highest biomasses in the highest trophic levels i.e. the producers are small organisms with least biomass and the biomass gradually increase towards the top of the pyramid. It is due to higher reproducibility and shorter lifespan of phytoplanktons, that although their biomass is less at any time, they frequently replenish themselves to meet the increased needs of zooplankton and larger fish. The increase of prey growth rate, the conversion efficiency, the prey carrying capacity, or the predator life span robustly facilitates the development of inverted biomass pyramids^41^. In the sCCLME, no significant trend was observed for the PA barycenter for all trophic groups. This absence of spatial shift in PA might be related to the high productivity of the sCCLME^2^. Future advances in remote technologies are expected to facilitate the greater discrimination of pelagic organisms^42^.

The geographic range and abundance of pelagic species are strongly associated with the temperature tolerance of organisms (thermal window)^43,44^. Temperature is a key driver of changes to communities at different spatial scales. Increases in temperature lead to the dominance of species adapted to warmer waters in the pelagic community^45^. Out of the most important species in the sCCLME, two categories emerged based on their temperature preferences: (1) cold water species, such as *Sardina pilchardus*, *Scomber colias,* and *Trachurus trachurus*, and (2) warm water fishes, such as the *Sardinella aurita, Sardinella maderensis,* and *Trachurus trecae*^46^. The abundance of *S. aurita*, a dominant pelagic fish species in the sCCLME, responded to warming. Low catches of *S. aurita* were associated to temperatures below 21 °C in the waters off Mauritania, whereas high catches were associated with temperatures above 21 °C in the waters off Senegal^12,47,48^. Range shifts in marine species linked to SST warming have been documented across all ocean regions^7^. Sarré et al.^49^ reported northward shifts in the distribution of sardinella and other small pelagic species in the sCCLME, which contrasted with our results, despite falling within the same study period. The authors attributed the northward shift of these low thermal tolerance species to high warming trends in the southern part of sCCLME. The population dynamics and physiological rates of zooplankton are strongly linked to temperature^14,50^. In the north-east Atlantic, the distribution of two species of copepods (*Centropages chierchiae* and *Temora stylifera*) shifted northwards in response to rising sea temperature^51,52^. This northward shift of copepod assemblages in response to global warming was estimated to be around 260 km per decade^50^.

The PA of PG and PFG was higher in the area to the south versus north of Cap Blanc, which was attributed to oceanographic conditions, particularly Chl-*a*^53^. The area south of Cap Blanc was characterized by significant SST warming associated with a stable UW, which provided a favorable habitat for pelagic species^7^. Moreover, previous studies^54^ showed that marine organisms aggregate as dense layers of PA under stable physical conditions. In our case, the area north of the Cap Blanc was characterized by high levels of UW, which did not favor the presence of a dense PA layer.

Although, previous studies^49^ in North West Africa demonstrated a northward shift of *S. aurita*, which is a key PFG species, other pelagic fish species exhibited phenotypical adaptations to changes in temperature^55^. Many fish species have geographic ranges spanning a wide temperature gradient, suggesting a capacity for acclimation or adaptation to changing temperature^56^. Indeed, temperature is a limiting factor to fish species with tropical affinity, but not to species with wider temperature tolerance. For example, *S. maderensis*, which tolerates high changes in temperature^47^, did not exhibit a northward shift^49^. However, oceanographic surveys are typically implemented in November–December during the southward migration of *S. aurita* from Morocco to Senegal^44^. This phenomenon might partly explain the higher PFG PA detected to the south of Cap Blanc in the current study. The few studies investigating the response of marine fishes to climate change in the sCCLME have focused on fish, and usually single species, rather than pelagic organisms as a whole. Yet, the population dynamics of zooplankton are tightly linked to temperature, which exhibit rapid responses to changing environments. Zooplankton species also respond to SST warming by shifts in their distribution^51,52^ and phenology^57^, or by changing their morphological traits, such as reducing body size^58,59^.

Relative spatial coherence was documented between environmental and PA anomalies (Fig. S5) underlying the complex relationship between PA and their environment. The only links found by comparing PA with environmental anomalies were: (1) the negative anomaly of PFG PA in 2006, coinciding with SSC, and (2) the negative anomaly of PFG PA in 2011, coinciding with that of SST. The noticeable increase of PFG NASC documented in the area south of Cap Blanc in 2015 (paralleled by a 50% drop in the north in the same year) could not be attributed to any environmental change, as no environmental anomaly was observed.

The adaptive DVM behavior^60,61^ has been reported, and was higher for PG, which had high Δ_DVM_. Small pelagic fishes disperse widely at night and aggregate by day with small bathymetric changes^62^. In comparison, planktonic organism performs true DVM; specifically, they occupy deep waters during the day and migrate toward the surface at night to feed^63^. Such behaviors explain the higher extent of DVM (i.e., high Δ_DVM_) reported for PG. In the area north of Cap Blanc, we documented higher PA during day compared to at night for PG in the upper water column (20–40 m). This phenomenon indicates an inverse DVM behavior, whereby plankton ascend in at dawn and descend at dusk. This inverse DVM pattern, named DVM type II, was previously documented for planktonic fish larvae in the sCCLME^63^. The more marked DVM (higher positive Δ_DVM_) in the south of Cap Blanc suggests that this area is mostly populated by communities performing DVM, particularly PG. Yet, the absence of a clear DVM trend allow to confirm that key functional pelagic groups remain stable in system over the study period.

Resistance is the ability for an ecosystem to remain unchanged when being subjected to a disturbance or disturbances. The absence of significant long-term trends in PA for both trophic level groups (PG and PFG), and absence of longitudinal shifts, demonstrates the strong resistanceof the sCCLME ecosystem to environmental stressors (overfishing, climate change). Indeed, despite the well-known overfishing statute of the main pelagic fish stocks and environmental change documented in the sCCLME, particularly for SST and UW shifts, no trend emerged for long-term variation in PG or PFG PA and DVM behavior. The sCCLME, particularly in the area south of Cap Blanc, presented strong inter-annual environmental variability, which did not impact long-term variation in PG and PFG. Moreover, as a tropical system, the sCCLME is characterized by high species diversity^64^, which increases system resistance. Diversity, connectivity, and adaptive capacity represent ecological properties that underlie ecosystem resilience^65^. Biological diversity in sCCLME might contribute towards resistance by increasing the likelihood that some species and/or functional groups are resistant to stressors. This phenomenon allows species to compensate for one another within a community, and facilitates ecological processes vital for recovery and adaptation. The phenotypic plasticity of key species in the PFG, such as *S. aurita*^55,66^ and *S. maderensis*^47^, to the changing climate facilitates the rapid evolution of traits better suited to new conditions.

This work presents a first step in understanding the resistance of the sCCLME at the pelagic level. Future studies are encouraged to investigate the ecological responses of the sCCLME to the effects of climate change at individual, population, and community levels.

## Methods

### Survey area

The research vessel R/V Dr. Fridtjof Nansen (DFN) conducted regular surveys in the marine area off West Africa. We focused on data collected from the southern part of the CCLME (hereafter named sCCLME; Fig. 1). Cap Blanc (20.77°N) is considered to represent a “faunistic limit” for the planktonic population^63^, and the northernmost distributional area for several small pelagic fishes in North-West Africa^67^. Thus, we divided our surveys in the sCCLME into two parts: area north of Cap Blanc *vs*. area south of Cap Blanc, extending from Cap Cantin (31° 65′ N) in Morocco to Casamance (12° 31′ N) in southern Senegal, respectively.

The sCCLME is rich in zooplankton, with copepods dominating. Copepod species account for 60 to 95% of total zooplankton abundance in the sCCLME, and constitute the bulk of PA of macro and mesozooplankton^68,69^. At a higher trophic level, the bulk of pelagic biomass is represented by small pelagic fishes (e.g., *S. aurita*, *S. maderensis*), which are also key for safeguarding food security in West Africa.

### Acoustics and environmental data processing

#### Acoustic data

Fisheries acoustic surveys were performed annually in November or December (upwelling season) from 1995 to 2004, and again in 2006, 2011, and 2015 (Fig. 1), on-board the research vessel R/V Dr. Fridtjof Nansen (DFN). The way in which total acoustic density is distributed divides the study area into two parts: north and south, separated by Cap Blanc. These surveys were conducted using a 38 kHz echosounder at depths ranging from 10 to 500 m isobaths. We deployed an ES38-B transceiver, towed at a depth of 5.5 m, with the following settings: absorption coefficient of 8.7 dB km^−1^, pulse length of 1.024 ms^−1^, and maximum used transmission power of 2000 W^70^. The echo sounder was calibrated following classic calibration procedures^71^. Acoustic data were processed with an offset of 10 m below the sea surface to avoid integrating air bubbles during post processing.

#### Environmental data

To obtain a homogenized overview of the area, environmental data were collected from satellite remote sensing products: upwelling wind (UW), sea surface chlorophyll-*a* concentration (SSC), and sea surface temperature (SST). UW is the upwelling-favorable meridian wind speed at the sea surface, and is used as an upwelling index^3^. UW was extracted from the daily CCMP (Cross-Calibrated MultiPlatform) wind product V2.0 at a 0.25° spatial resolution (available at http://www.remss.com/measurements/ccmp/) from 1988 to 2017. SSC is an adequate proxy of primary productivity models on which biomass is directly based^32^. SSC was collected daily from the SeaWIFS (1997–2002) and AQUA-MODIS sensor (2003–2018) (available at https://oceancolor.gsfc.nasa.gov/). SST data were extracted from daily day-time series of the pathfinder AVHRR dataset version 5.2, from 1982 to 2018, at 4 km resolution (available at https://www.nodc.noaa.gov/SatelliteData/pathfinder4km53/). The spatial averages of satellite data were computed using a fixed distance from the coast of 100 km. The monthly average of all data (UW, SSC, and SST) were calculated, and the spatial averages were computed for the sCCLME area. Annual climatological anomalies were estimated to elucidate fluctuations of the different environmental variables and support the trend analysis.

### Data analysis

Acoustic data were echo-integrated per 0.1 nautical miles (nmi) using ‘Matecho’^72^. To obtain homogenous data, only continental shelf data were considered (i.e., 10–150 m). The relative acoustic density, expressed as Nautical Area Scattering Coefficient (NASC or s_A_, m^2^ nmi^−2^)^73^, was used as a proxy of marine organism abundance to assess spatial variability. In the sCCLME area, the abundance of small pelagic fish is closely correlated to the upwelling of nutrient-rich waters, which is the basis of food production for these species^2,48^. Given that small pelagic fish mainly feed on phyto- and zooplankton, which are very sensitive to environmental changes, changes to primary production might affect the abundance of stocks and, thus, both the structure and functioning of pelagic ecosystems. Consequently, a “thresholding” approach was applied, using volume backscattering strength (S_v_, expressed in dB), to separate marine organisms into three echo classes: “low acoustic trophic level” [− 80 ≤ S_v_ < − 65 dB]^74,75^, “high acoustic trophic level” [− 65 ≤ S_v_ < − 20 dB]^76–78^ and the “all trophic level” [− 80 ≤ S_v_ < − 20 dB]. Low and high acoustic trophic level classes were assumed to represent plankton (i.e., meso and macrozooplankton) *vs*. pelagic fish, respectively. Usually, small pelagic fishes occurring in the CCLME have a S_v_, higher than ~ − 68 dB^79^, while macrozooplankton backscatter at < − 70 dB^16,80^. Low, high, and total acoustic trophic levels were, thus, designated as plankton (PG), pelagic fish group (PFG), and total group (TG), respectively.

To detect spatial changes in the spatial distribution of pelagic marine organisms in the areas north and south of Cap Blanc, we analyzed the barycenter displacements of PA over the time-series. The barycenter is the average position of acoustic density for 1 year, weighted by the biomass of each zone. For instance, if “B_ai_” represents the biomass in zone “Z_i_” for year “a” and “A_i_” the coordinates of the center of “Z_i_”, the coordinates of the barycenter “C_a_” of the biomass would fit the equation below as:

1 $$\sum \limits_{i=1}^{n} {B}_{ai}\overrightarrow{{C}_{a}{A}_{i}}={B}_{a1}\overrightarrow{{C}_{a}{A}_{1}}+ {B}_{a2}\overrightarrow{{C}_{a}{A}_{2}}+\dots + {B}_{an}\overrightarrow{{C}_{a}{A}_{n}}=\overrightarrow{0}$$

And the coordinates in latitude “*X*_*a*_*”* and longitude “*Y*_*a*_*”* of C_a_ are:

2 $${X}_{a}=\frac{{\sum }_{i=1}^{n}{B}_{ai}*{X}_{ai}}{{\sum }_{i=1}^{n}{B}_{ai}} \quad {Y}_{a}=\frac{{\sum }_{i=1}^{n}{B}_{ai}*Yai}{{\sum }_{i=1}^{n}{B}_{ai}}$$

All statistical analyses were completed in a free software environment for statistical computing and graphics^81^. Figures and maps were also performed with R and Matlab R2018. Spatial and temporal variability of the acoustic abundance time-series were investigated for: “zooplankton,” “pelagic fish,” and “total.” The statistical significance of the annual position (latitude and longitude) of changes to the barycenter and PA trend were tested using Spearman tests, which are considered valid for testing the significance of trends^82^. The Spearman rank correlation can be used to determine if a variable is increasing or decreasing with time. Spearman's rho is the product-moment correlation between the ranks of paired data. To test for trend, one member of the pair is the time of observation, the other is the observed variable^83^ Trends in the annual means of environmental variables (i.e., SST, UW, and SSC) were assessed in the north and south of Cap Blanc using Spearman tests, which are valid for supplementing linear models with a non-parametric models, such as the Spearman correlation. Diel changes to PA in the sCCLME were first analyzed by comparing PA (log_10_ transformed) for day vs night in the vertical dimension (i.e., the mean value for each 1-m depth step over the whole period study). Then, Δ_DVM_ (night PA–day PA) was computed for each 1-m depth step and each year. Diel change in the annual PA for each acoustic group was tested using the Wilcoxon test. The Spearman test was used to assess DVM trends for PG and PFG in the north and south of Cap Blanc. Diel transition periods were removed from the analyses to prevent diel vertical migrations causing bias to changes in acoustic density. Transition periods were defined using sun altitude, with sunset and sunrise corresponding to a sun altitude of 18° and + 18°, respectively. Time series anomalies for environmental variables and acoustic groups were calculated by computing differences between actual (annual or monthly) and long-term average values.

## Supplementary Information

Supplementary Information.

## Data Availability

The public cannot access our acoustic data because they belong to the partners who funded the oceanographic cruise. Environmental data are already available as indicated in the methodology part.
