# Author Correction: On the robustness of an eastern boundary upwelling ecosystem exposed to multiple stressors

**DOI:** 10.1038/s41598-021-88183-x

**Published:** 2021-04-13

**Authors:** Ndague Diogoul, Patrice Brehmer, Hervé Demarcq, Salaheddine El Ayoubi, Abou Thiam, Abdoulaye Sarre, Anne Mouget, Yannick Perrot

**Affiliations:** 1 Institut Sénégalais de Recherches agricoles (ISRA), Centre de Recherches Océanographiques de Dakar-Thiaroye (CRODT), Dakar, Senegal; 2 IRD, CNRS, Univ Brest, Ifremer, DR Ouest, Lemar, Plouzané, France; 3 IRD, IFREMER, CNRS, Univ Montpellier, Marbec, Sète, France; 4 Institut National de Recherche Halieutique INRH, Agadir, Morocco; 5 University Cheikh Anta Diop (UCAD), Institute of Environmental Science (ISE), BP 5005, Dakar, Senegal

Correction to: *Scientific Reports* 10.1038/s41598-021-81549-1, published online 21 January 2021

The Acknowledgements section in this Article is incomplete.

“This work was completed within the AWA “Ecosystem Approach to the management of fisheries and the marine environment in West African waters” project funded by the IRD and BMBF (grant 01DG12073E and 01DG12073B), http://www.awa.ird.fr (SRFC: Sub Regional Fisheries Commission) and the PREFACE project funded by the European Commission’s Seventh Framework Program (2007–2013) under Grant Agreement number 653521, https://preface.b.uib.no/ and ended within TriAtlas European project (grant agreement number 817578). We thank the Nansen project (FAO/IMR), Dr. Jens-Otto Krakstad, and IMR Norway as FAO and West African colleagues involved in data collection.”

should read:

“This work was completed within the AWA “Ecosystem Approach to the management of fisheries and the marine environment in West African waters” project funded by the IRD and BMBF (grant 01DG12073E and 01DG12073B), http://www.awa.ird.fr (SRFC: Sub Regional Fisheries Commission) and the PREFACE project funded by the European Commission’s Seventh Framework Program (2007–2013) under Grant Agreement number 653521, https://preface.b.uib.no/ and ended within TriAtlas European project (grant agreement number 817578). We thank the Nansen project (FAO/IMR), Dr. Jens-Otto Krakstad, and IMR Norway as FAO and West African colleagues involved in data collection. N.D. gratefully acknowledges the funding received towards her PhD from the Organisation for Women in Science for the Developing World (OWSD) and The Swedish International Development Cooperation Agency (SIDA).”

