# Supplementary material for: On the robustness of an eastern boundary upwelling ecosystem exposed to multiple stressors
Source: Sci Rep. 2021 Jan 21;11:1908. doi: 10.1038/s41598-021-81549-1 (PMC7819996; doi:10.1038/s41598-021-81549-1)
Supplement: Supplementary file 1 — Supplementary Information. [file 41598_2021_81549_MOESM1_ESM.pdf]

On the resilience of an east boarder upwelling ecosystem exposed to multiple stressors

Ndague DIOGOUL[1,2,5, *], Patrice BREHMER[1,2], Hervé DEMARCQ[3], Salaheddine EL AYOUBI[4], Abou THIAM[5], Abdoulaye SARRE[1], Anne MOUGET[2] and Yannick PERROT[2]

[1]Institut Sénégalais de Recherches agricoles (ISRA), Centre de Recherches Océanographiques de Dakar-Thiaroye (CRODT), Dakar, Senegal

[2]IRD, Univ Brest, CNRS, Ifremer, DR Ouest, Lemar, Plouzané, France

[3]IRD, IFREMER, CNRS, Univ Montpellier, Marbec, Sète, France

[4]Institut National de Recherche Halieutique INRH, Agadir, Morocco

[5]University Cheikh Anta Diop (UCAD), Institute of Environmental Science (ISE), BP 5005, Dakar, Senegal

Corresponding author: *diogoulndague@yahoo.fr

Supplementary material

Fig. S 1: Monthly means and 13-term smoothing average of sea surface temperature (SST, °C), and upwelling wind (UW, m s^{-1}) in the areas north and south of Cap Blanc. SST and UW shift are delimited by a black line. The periods covered by the biological data (1995 to 2004, 2006, 2011, and 2015) are shaded in grey.

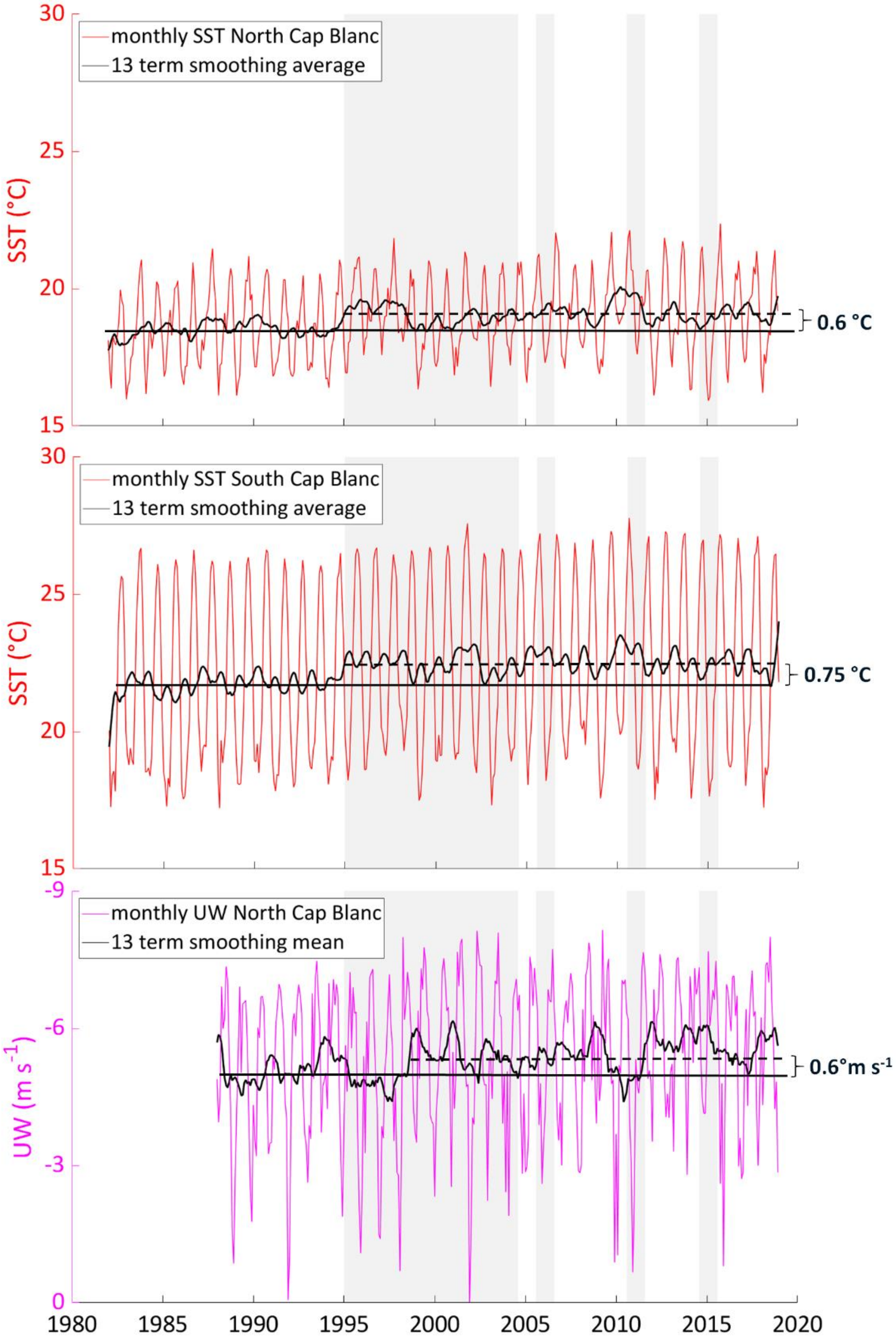

Fig. S 2: Variation in the mean Nautical Area Scattering Coefficient (NASC, m^2 nmi^{-2}) in the areas north and south of Cap Blanc for the plankton group (PG), pelagic fish group (PFG), and total group (TG) in North and South Cap Blanc.

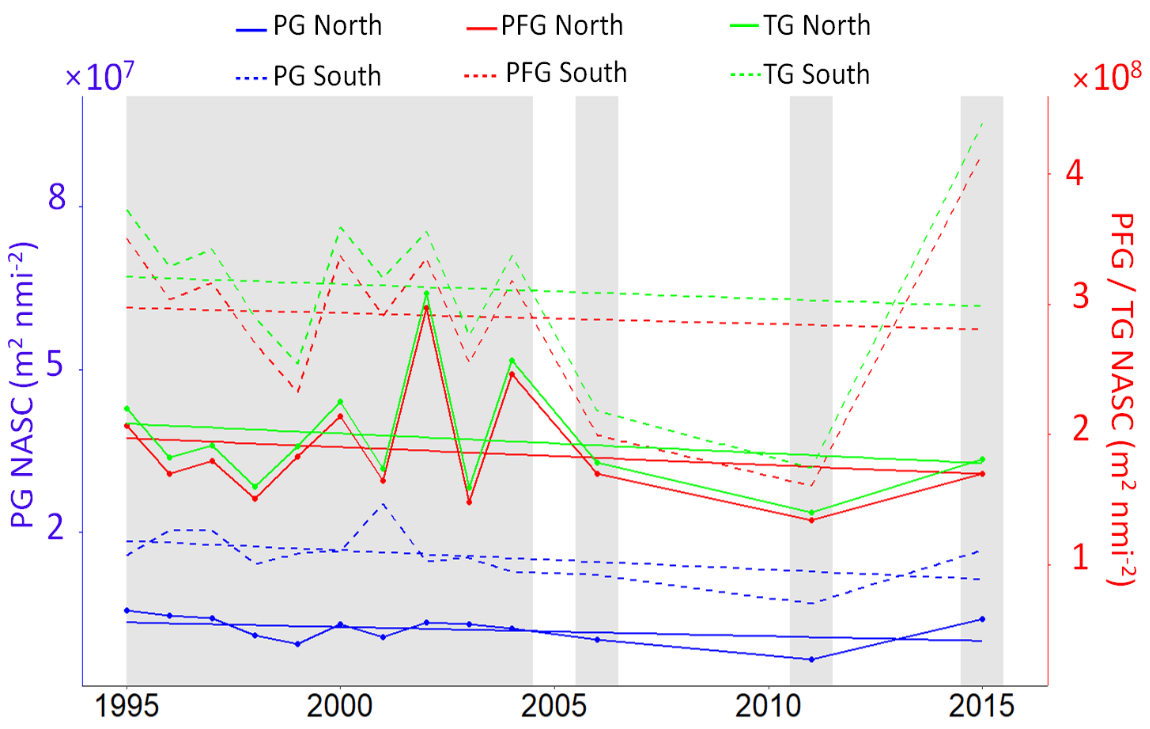

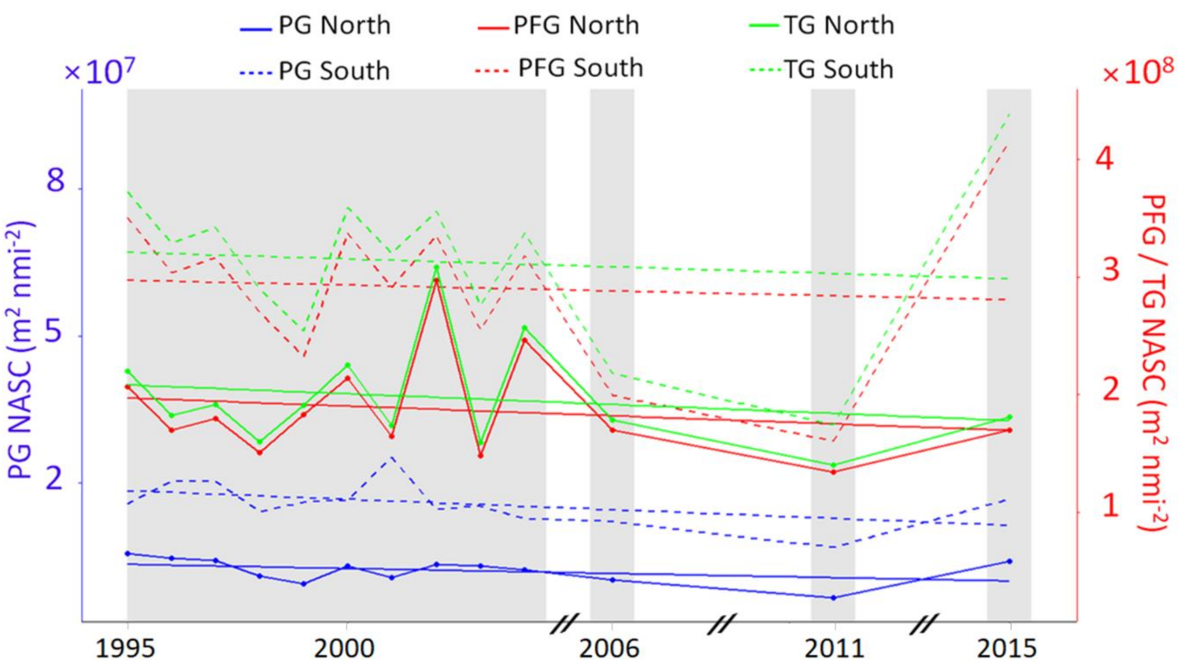

Fig. S 3: Anomaly in the mean relative pelagic biomass (expressed as Nautical Area Scattering Coefficient NASC, m² nmi^{-2}) in North and South Cap Blanc for total group (TG) in areas north and south of Cap Blanc.

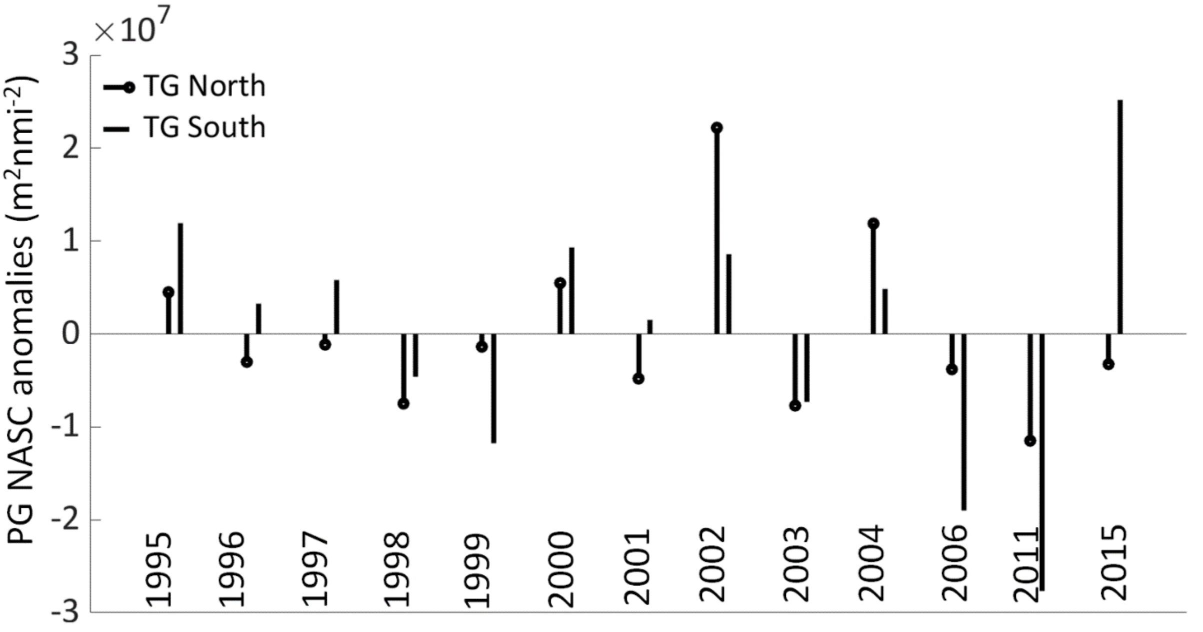

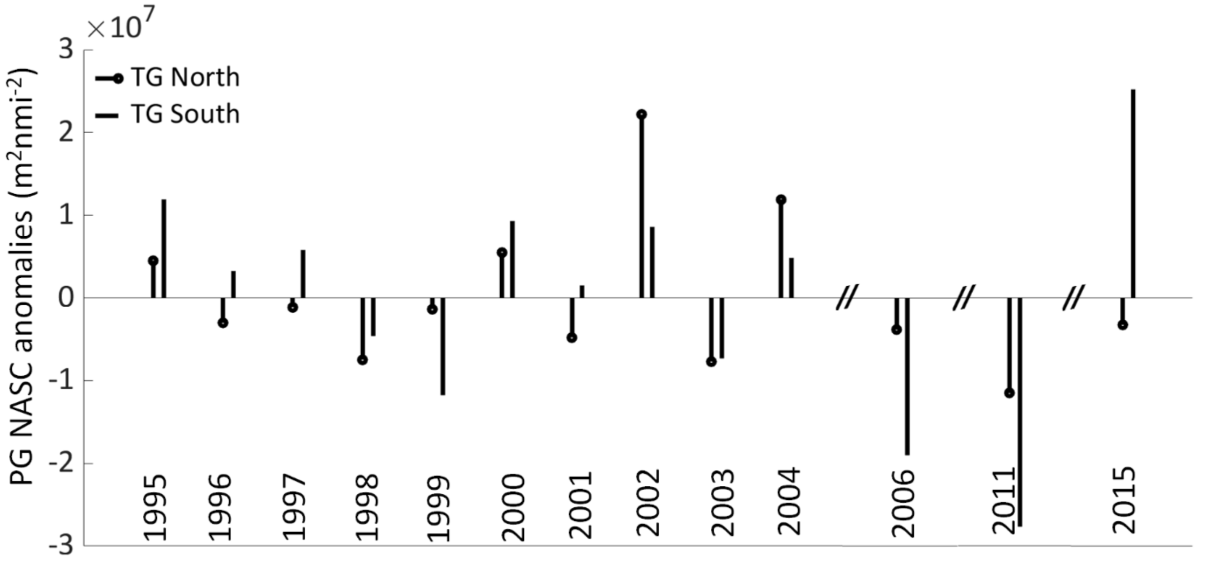

Fig. S 4: Box plot (minimum, maximum, and median) of relative pelagic biomass (expressed as Nautical Area Scattering Coefficient noted NASC, m² nmi m^{-2}) grouped by diel period (day and night) for the plankton group (PG), pelagic fish group (PFG), and total group (TG) in the area north and south of Cap Blanc. Wilcoxon test comparing the mean between day and night was significant (*p*-value < 0.001) for each group.

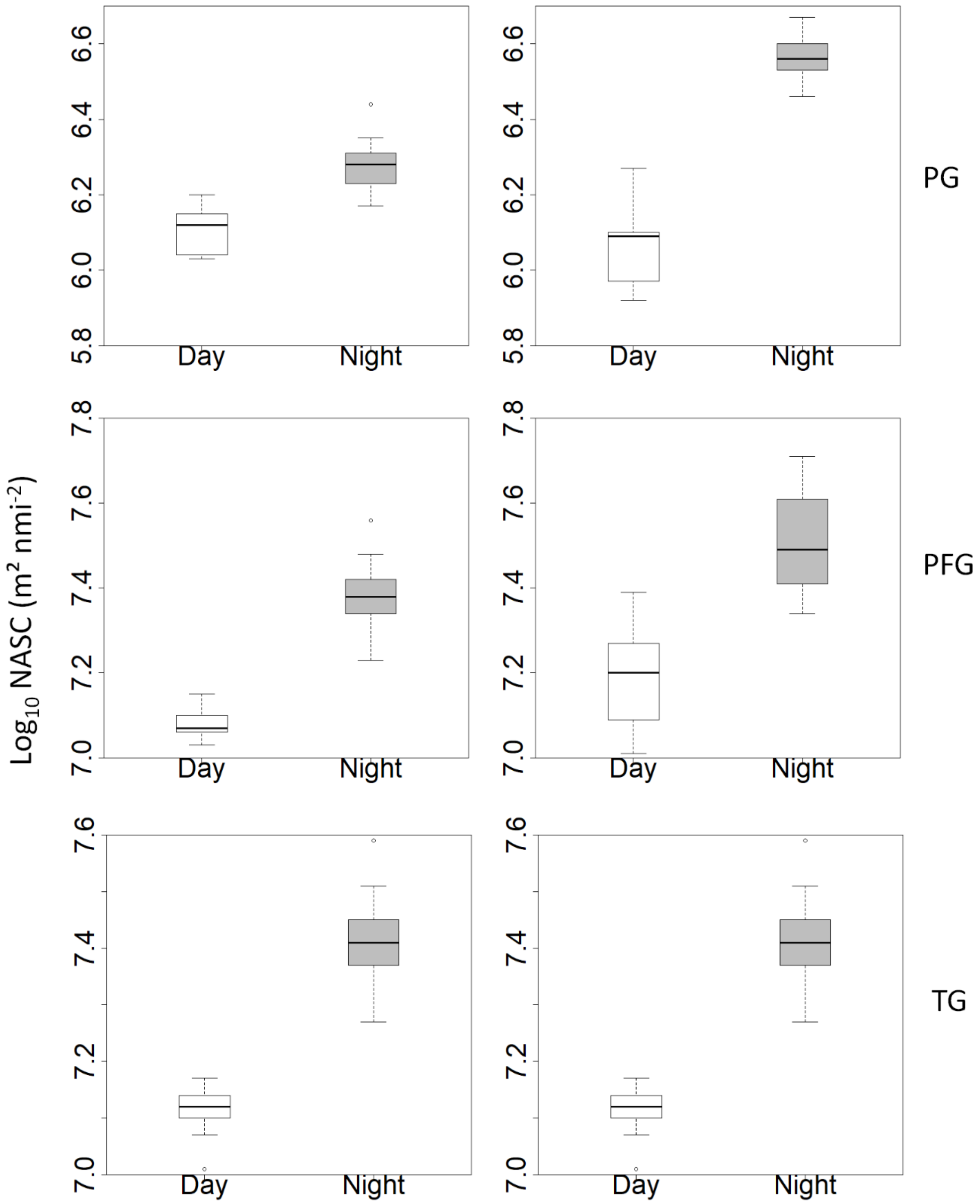

Fig. S 5: Comparison of the plankton group (PG) and pelagic fish group (PFG)) with respect to relative pelagic biomass (expressed in Nautical Area Scattering Coefficient noted NASC, m² nmi m^{-2}) anomalies and environmental anomalies (sea surface temperature (SST), favorable upwelling wind (UW), and sea surface chlorophyll-*a* concentration (SSC)) in the area north of Cap Blanc (left) and south of Cap Blanc (right) in the southern part of the Canary Current Large Marine Ecosystem.

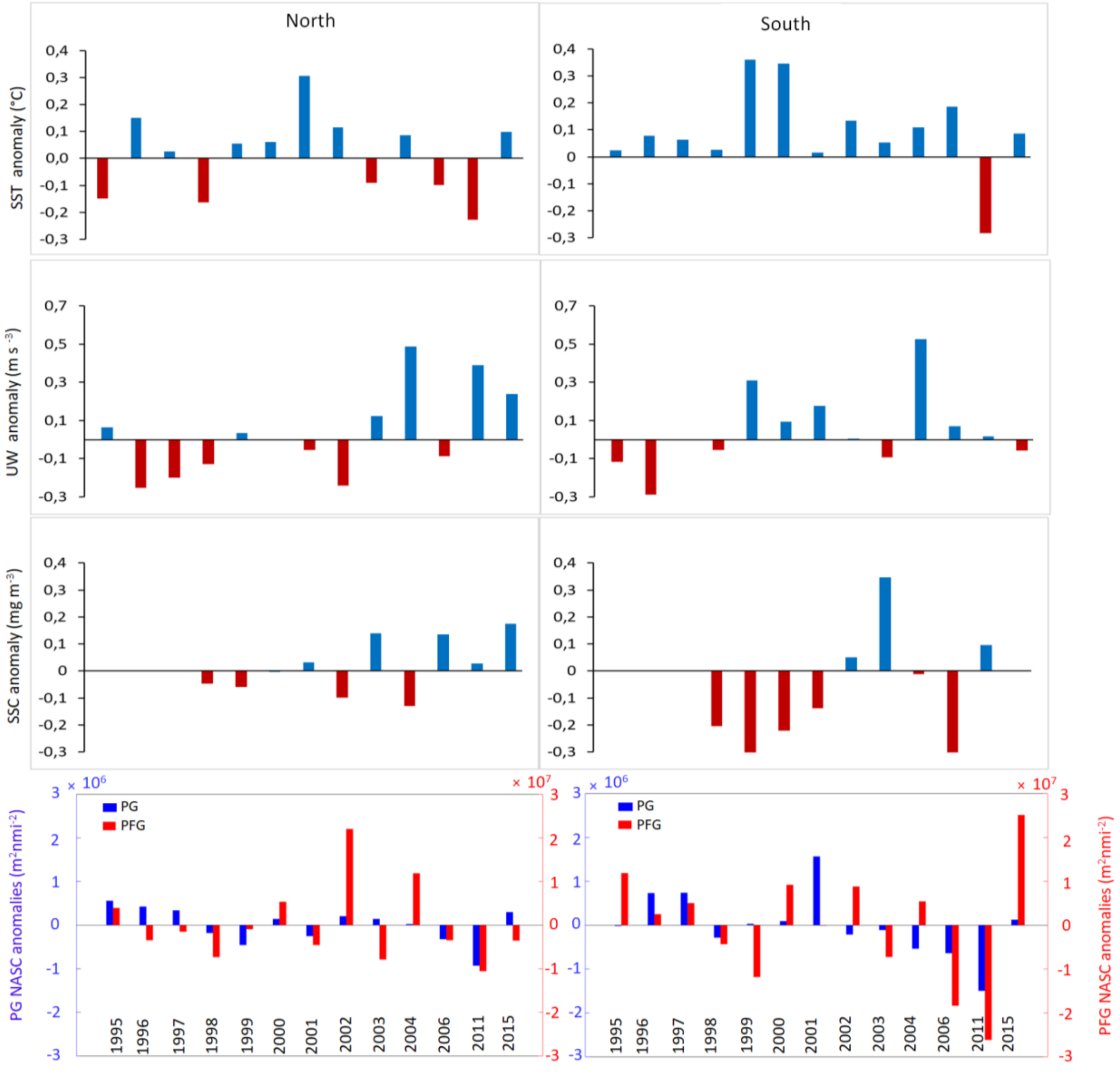

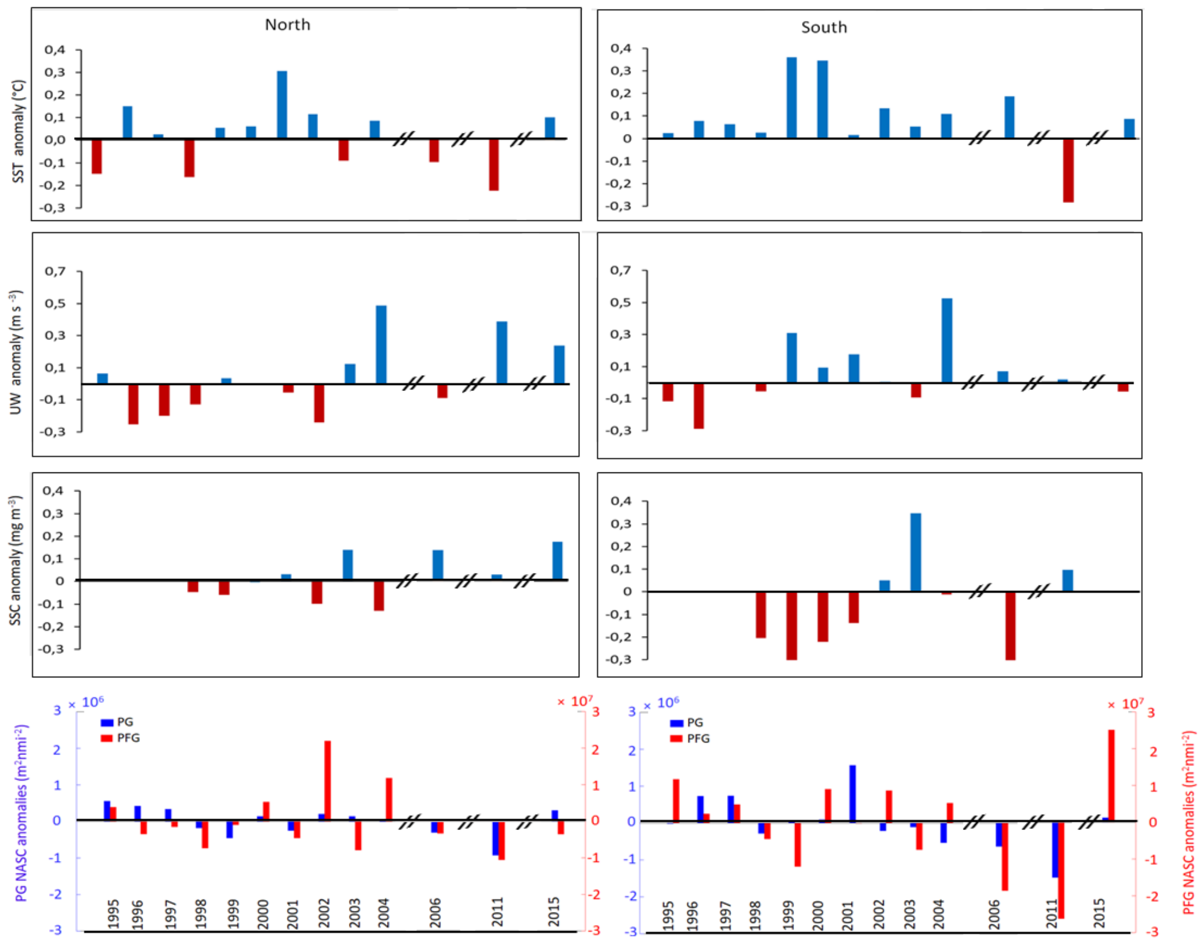
North
South
SST anomaly (°C)
UW anomaly (m s-1)
SSC anomaly (mg m-3)
PG NASC anomalies (m²nmi⁻²)
PFG NASC anomalies (m²nmi⁻²)
× 10⁶
× 10⁷
PG
PFG
1995
1996
1997
1998
1999
2000
2001
2002
2003
2004
2006
2011
2015
